# The Writing of Galen

**Published:** 1884-08

**Authors:** 


The Writing of Galen.—The *Amer. Practitioner* says: Most of us have but a faint conception of the vast extent of the writing of Galen. In a sketch of his life by Dr. G. J. Fisher, in the *Annals of Anatomy and Surgery*, as the result of a careful comparative computation, it was found that the united lines of the Bible amount to sixteen thousand feet, while those of a Greek edition of Galen extended to eighty-seven thousand feet, or sixteen and a half miles, exclusive of all headings, notes, and references. It is five times more voluminous than the entire Bible. But this represents only his extant works. A portion of his manuscripts were destroyed by fire, and not a few by the lapse of time. It is supposed that he wrote no less than five hundred treatises, of which one hundred and sixty-eight are supposed to be lost. He wrote not alone on medicine, but on mathematics, logic, and philosophy. Most of his writings were in Greek, elegant in style, but prolix and abounding in conceit, yet fluent, "fresh and familiar as if spoken yesterday."

